# Naringenin modifies T-helper responses and macrophage activities in BALB/c mice

**DOI:** 10.22038/AJP.2023.23382

**Published:** 2024

**Authors:** Fatemeh Keivan, Seyyed Meysam Abtahi Froushani

**Affiliations:** *Department of Microbiology, Faculty of Veterinary Medicine, Urmia University, Urmia, Iran*

**Keywords:** Naringenin, Prednisolone, Ani-inflammatory, Immunomodulation, Immunosuppression

## Abstract

**Objective:**

Naringenin is a naturally occurring flavonoid found in citrus fruits. This study was done to compare the oral immunomodulatory effects of naringenin and prednisolone.

**Materials and Methods:**

The effect of one-month oral administration of naringenin (10, 20, and 40 mg/kg) and prednisolone (2 mg/kg) on peritoneal macrophage was compared in the first set of experiments. Separate evaluations were conducted on the effects of naringenin on* in vivo* and *ex vivo*T-helper (T_h_) lymphocyte responses and their subsets in mice immunized with ovalbumin (OVA). Animals challenged with OVA received oral doses of naringenin or prednisolone from two days prior to immunization to 28 days after immunization.

**Results:**

Naringenin and prednisolone increased macrophages’ respiratory burst, and nitric oxide and interleukin (IL)-10 production while decreasing IL-12 production. Macrophages isolated from mice administered with 40 mg/kg naringenin had greater phagocytic potential than those isolated from mice administered with prednisolone. OVA-challenged mice treated with 40 mg/kg naringenin or prednisolone had decreased delayed-type hypersensitivity comparable to control mice. The splenocyte proliferation index was lower in the prednisolone-treated group than the naringenin-treated group, even at 40 mg/kg. In the splenocyte cultures, both agents decreased *T-bet *expression. Naringenin, in contrast to prednisolone, did not affect *GATA3*expression. The 40 mg/kg naringenin dose reduced *RORγt* more effectively than prednisolone.

**Conclusion:**

All these findings indicate the potential of naringenin as a modifying agent of immune responses. Consequently, naringenin may be beneficial in controlling some immunopathological conditions.

## Introduction

Immunopathological conditions initiate a series of cellular responses that are followed by tissue damage and an inflammatory response. The damaged tissue will progress toward healing and repair if sufficient homeostasis mechanisms are established. (Etemadi et al., 2022; Oishi and Manabe, 2018). Chronic and unresolved inflammation causes significant tissue damage and dangerous complications (Etemadi et al., 2022). Immunosuppressive drugs such as glucocorticoids, methotrexate, cyclosporine A, rapamycin, and cyclophosphamide are widely used today to treat autoimmune diseases or prevent rejection of transplanted tissues (Golbahari and Abtahi Froushani, 2019; Parandin et al., 2023; Sobotková and Bartůňková, 2019). However, in addition to their limited therapeutic benefits, many of these drugs have dangerous side effects such as increased risk of dangerous infections, hepatotoxicity, diabetes, and insomnia (Sobotková and Bartůňková, 2019). Consequently, it is necessary to find new compounds that modulate and regulate immune system responses rather than simply suppressing them. In recent years, scientific sources have focused on using herbal medicinal products to find new pharmacological substances (Golbahari and Abtahi Froushani, 2019). 

Flavonoids exhibit powerful antioxidant and anti-inflammatory properties (Panche et al., 2016; Tungmunnithum et al., 2018). Naringenin (5,7-Dihydroxy-2-[4-hydroxyphenyl] chroman-4-one) is one of the most famous and abundant flavonoids found in *Citrus* species (such as grapefruit, orange, and bergamot (Erlund et al., 2001; Hajizadeh et al., 2021). Blood and intracellular levels of naringenin can rapidly increase following ingestion of this flavonoid (Erlund et al., 2001). The antioxidant, antiviral, anti-inflammatory, anti-allergic, anti-hepatotoxic, anti-cancer, anti-diabetic, anti-hyperlipidemia, anti-obesity, and anti-coagulant properties of naringenin have been reported (Hajizadeh et al., 2021). There is substantial evidence that naringenin affects controlling and reducing symptoms of disorders caused by improper regulation of the immune system, including experimental autoimmune encephalomyelitis (EAE), rheumatoid arthritis, psoriasis, and ankylosing spondylitis (Deenonpoe et al., 2019; Hajizadeh et al., 2021; Liu et al., 2016; Niu et al., 2021). Intriguingly, several studies report the advantages of naringenin against SARS-CoV-1 and MERS (Clementi et al., 2021). Complications of both diseases result from an abnormal response of T-helper (T_h_) lymphocytes and innate cells, such as macrophage lineage (Boechat et al., 2021). Therefore, it is reasonable to assume that some of the beneficial effects of naringenin in these diseases are due to its outcomes on Th cells and macrophages.

Macrophages can be reprogrammed in response to their microenvironment, leading to the emergence of distinct functional phenotypes such as classically activated and alternatively activated macrophages (Cutolo et al., 2022). T lymphocytes can polarize and form distinct functional patterns depending on their microenvironment. The most well-known patterns of polarization of T lymphocytes include Th1 (signature cytokines interferon gamma (IFN-γ) and interleukin 2 (IL-2), Th2 (signature cytokines IL-4 and IL-5), and Th17 (signature cytokines IL-17A and IL-22), and T-regulatory (Treg) lymphocytes (signature cytokines IL-10 andtrans forming growth factor-beta (TGF-β) (Tuzlak et al., 2021). Several destructive immune diseases are caused by an imbalance between Th cell subsets and macrophage subsets. Consequently, inappropriate polarization of Th lymphocytes or macrophages forms the basis of immune dysregulation disease (Bart et al., 2021). 

According to previous research, flavonoids may alter the function of macrophages or lymphocytes (Ehrchen et al., 2019; Hosseinzade et al., 2019). However, there is no well-founded and classical research on the immune-modifying effect of daily consumption of naringenin. Also, a comparison between the immune-modifying benefits of naringenin and a known immune-modifying drug such as prednisolone is not available. In this regard, the purpose of this study was to compare the effects of naringenin and prednisolone on *in vivo* and *ex vivo* Th lymphocyte responses and their subsets in ovalbumin-immunized mice. Ovalbumin (OVA) is a non-toxic, non-reactive T lymphocyte-dependent antigen commonly employed as a model protein for studying antigen-specific immune responses in mice. In a separate series of experiments, the impact of oral administration of naringenin and prednisolone for one month on the function of peritoneal macrophages was compared.

## Materials and Methods

**Chemicals**

Naringenin was procured from Chengdu Kanghui Bio-technology (China). Prednisolone was purchased from Iran Hormone Company (Iran). RNX-Plus solution for total RNA isolation was purchased from Sinaclon (Tehran, Iran). SYBR Premix Ex Taq II and cDNA reverse transcription kit were obtained from TAKARA (China). The enzyme-linked immunosorbent assay (ELISA) kits were purchased PeproTech EC, Ltd. (London, UK). Ovalbumin and other reagents were obtained from Sigma-Aldrich Corporation (St. Louis, MO, USA).

**Animals**

Male BALB/c mice (6- to 8 weeks old) were obtained from the Pasteur Institute of Iran. During the study, the animals were housed at 23°C with a light/dark cycle of 12 light to 12 hr dark and they had free access to standard laboratory food and water. All animal care and experiments protocols adhered to the Iranian Ministry of Health regulations and the Helsinki Convention on Experiments on Laboratory Animals.

**Evaluation of** **macrophage** **function after receiving naringenin**

Briefly, BALB/c mice were randomly divided into the following five groups (n=5): Control group (Control): The mice orally received Phosphate-buffered saline (PBS), prednisolone-treated mice (Pred.): Mice in this group were treated with a dose of 2 mg/kg prednisolone, naringenin-treated groups: The animals of these groups were orally treated with doses of 10 (NAR10), 20 (NAR20) and 40 (NAR40) mg/kg of naringenin. Orally administered prednisolone or naringenin was administered for 30 consecutive days. At the end of this step, the resident macrophages in the peritoneal cavity of mice were isolated by injecting ice-cold PBS, as per the literature (Pineda-Torra et al., 2015; Rios et al., 2017). The mice were euthanized following an intraperitoneal injection of 100 mg/kg ketamine and 10 mg/kg xylazine. In brief, 5 ml of ice-cold PBS was injected into the peritoneal cavity of mice. The peritoneal fluid was aspirated and centrifuged (600 g) for ten minutes at 4˚C. The cells were rinsed and re-suspended in RPMI-1640 medium supplemented with 10% heat-inactivated fetal calf serum. After counting cells with trypan blue dye, 100 μl of live cell suspension (2×10^6^ cells/ml) was incubated in 96-well microplates for 40 min at 37°C and 5% CO_2_. During this period, macrophages adhered to the plate. The non-adherent lymphocytes were omitted by intensively by rinsing with ice-cold PBS. The viability of macrophages examined with trypan blue exclusion, never dropped below 95%. 

The macrophages were incubated with 0.1% nitrobluetetrazolium (NBT) and 100 ng/ml tetradecanoylphorbol acetate (TPA) for 20 min to assess the potential for the respiratory burst of macrophages. The unexploited NBT dye was eliminated by rinsing. The reduced dye was solubilized in dioxin and monitored at 520 nm (Froushani and Galeh, 2014; Shushtari and Abtahi Froushani, 2017). The isolated cells were primed with LPS (100 ng/ml) for 6 hr to monitor the potential of nitric oxide production by macrophages. The cell-free supernatants were isolated and monitored through the Griess method (Shushtari and Abtahi Froushani, 2017). In short, the Griess reagent (0.1% naphthyl ethylenediamine, 0.1% sulfanilamide, and 3% phosphoric acid) and the supernatant were incubated for 10 min in the dark condition at room temperature. At the wavelength of 540 nm, the amount of optical density was reported. A standard curve was used to estimate the nitrite concentration.

The collected supernatant was also used to evaluate the levels of cytokines IL-10 and IL-12 via an ELISA method. The cells were also pulsed with neutral red-stained, heat-stabilized, zymosan suspension at a 1:10 ratio for 30 min to assess the phagocytic ability of macrophages. Afterward, the supernatants were removed, and the phagocytosis process was ceased by adding Baker’s formol calcium solution. The internalized neutral red was solubilized via acidified alcohol. The optical density was recorded at 550 nm (Shushtari and Abtahi Froushani, 2017).

**Naringenin administration and OVA immunization**

In a series of experiments, BALB/c mice were randomly divided into six groups (n=5) as follows: Control group (Control): The mice orally received Phosphate-buffered saline (PBS), OVA-immunized mice (OVA): Mice in this group were immunized with OVA according to the following protocol, OVA-Pred. group: This group included rats that received prednisolone (2 mg/kg) orally at the same time as immunization with OVA, naringenin-treated groups: The animals of these groups were immunized with OVA and orally received with doses of 10 (OVA-NAR10), 20 (OVA-NAR20) and 40 (OVA-NAR40) mg/kg of naringenin. Before the first immunization, animals were administered with the substances orally every other day for 28 days. For OVA-immunization, OVA (at 2 mg/ml in PBS) was emulsified in an equal volume of Complete Freund’s adjuvant (CFA), and then 0.1 ml of the emulsion was injected subcutaneously (SC) into the animals’ shaved backs. In addition, the mice were boosted ten days later with the same concentration of OVA as in the initial challenge in incomplete Freund’s adjuvant (IFA). A negative control (nonimmunized) group was injected SC with saline solution per the same schedule.

**Splenocyte proliferation**

The spleens of mice were aseptically isolated when the on the seventeenth day after immunization. Each spleen was crushed and passed through a wire mesh with a diameter of 20 μm. The isolated cell suspension was centrifuged at 2000 rpm for 10 min. Mononuclear cells were separated by a Ficoll–Hypaque density gradient. Red blood cells were deleted by ACK-RBC lysis buffer. A cell suspension (2×10^6^/ml in RPMI- 1640 medium supplemented with 10% fetal calf serum) was cultured in 96-well plates and stimulated with 100 µg/ml OVA for 72 hr. Afterward, wells were pulsed with 25 µl of the 3-(4,5-dimethylthiazol-2-yl)-2,5-diphenyltetrazolium bromide (MTT) solution (5 mg/ml). After 4 hr, 150 µl dimethyl sulfoxide was added to each well and shaken to solve the formazan crystals. The absorbance of cells stimulated with OVA was then measured at 550 nm, and the proliferation index was calculated by dividing the absorbance of stimulated cells with OVA by the absorbance of non-stimulated cells.

**Transcription factors expression in splenocytes**

Total RNA from the splenocyte population cultured with OVA or medium was extracted using the RNX-Plus solution according to manufacturer guidelines to analyze *Foxp3*, *T-bet*, *RORγt*, and *GATA3* expression. The extracted RNA was used to synthesize complementary DNA. The SYBR Green kit was used to perform PCR amplification in triplicate, per the manufacturer’s instructions. The *HPRT* gene was used as a reference gene. Sequences of forwarding and reverse primers for mRNA amplification are presented in Table 1. Final results are presented as relative fold change (RFC) against the nonimmunized control group values.

**Evaluation of** **delayed-type hypersensitivity**

In a separate set of experiments (identical treatment program as above), 50 µl OVA (1 mg/ml in equal volumes of PBS and IFA) was SC injected into the left hind paw on the twenty-eighth day after the start of the immunization. The same volume of PBS was injected into the rightfoot pad as a negative control. After 48 hr, footpad thickness was monitored by a digital caliper. The extent of the delayed-type hypersensitivity (DTH) was evaluated according to the following formula: [(Thickness of right footpad) _ (Thickness of left footpad] * 100/ (Thickness of left footpad) (Abtahi Froushani and Esmaili Gourvarchin Galeh, 2014).

**Statistical analysis**

All the statistical analyses were performed in MedCalc software (ver. 19). After confirming the normal distribution of the data using the Kolmogorov-Smirnov test, one-way analysis of variance (ANOVA) and Tukey’s test were used to compare the data. A p<0.05 was considered statistically significant. Data are expressed as mean±standard deviation (SD).

## Results

**Naringenin promoted a shift of responses towards the M2 phenotype**

**Figure 1:**
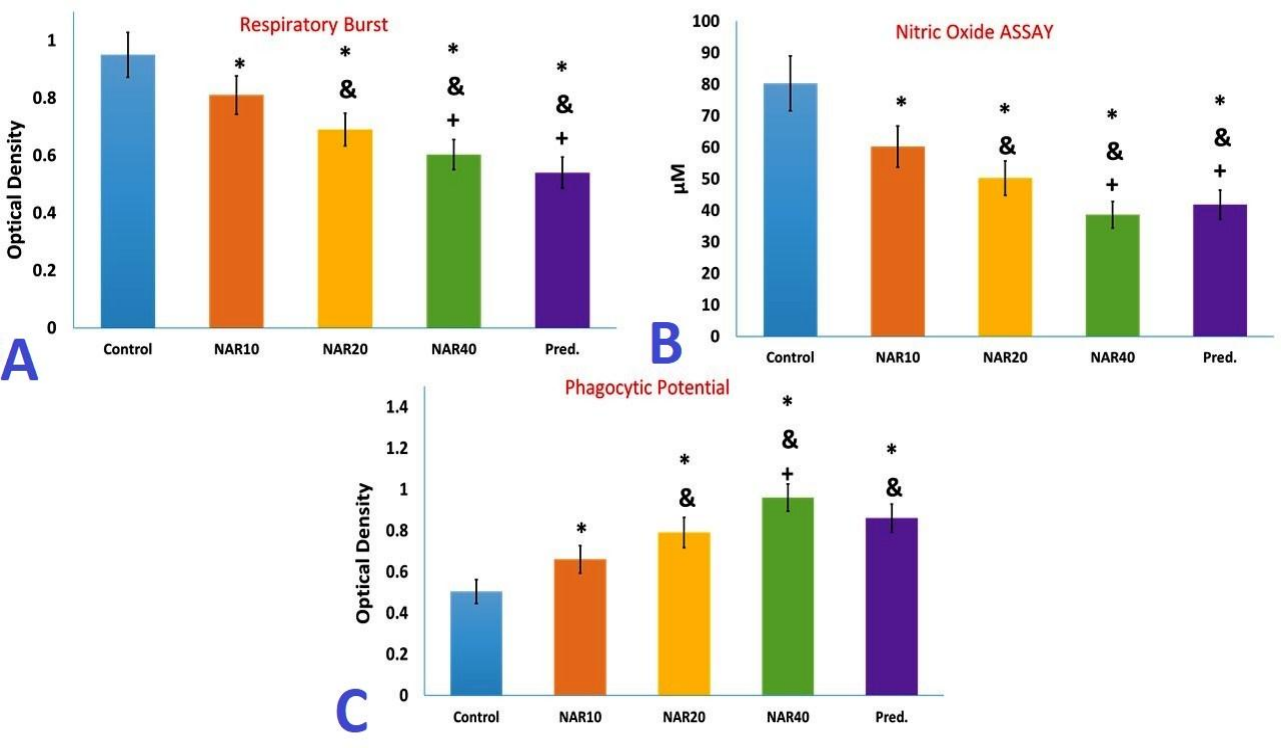
Evaluation of peritoneal macrophage. Following one month of oral treatment of Balb/C mice with naringenin or prednisolone, peritoneal macrophages were isolated and analyzed, as detailed under materials and methods. A) Assessment of macrophage respiratory burst after activation by tetradecanoylphorbol acetate. B) Nitric oxide (NO) production of macrophages after challenge with lipopolysaccharide. C) Evaluation of phagocytic potential of macrophages. The results are presented as mean±S.D. (* p<0.05 versus control mice, & p<0.05 versus NAR10 mice, + p<0.05 versus NAR20 mice)

**Figure 2:**
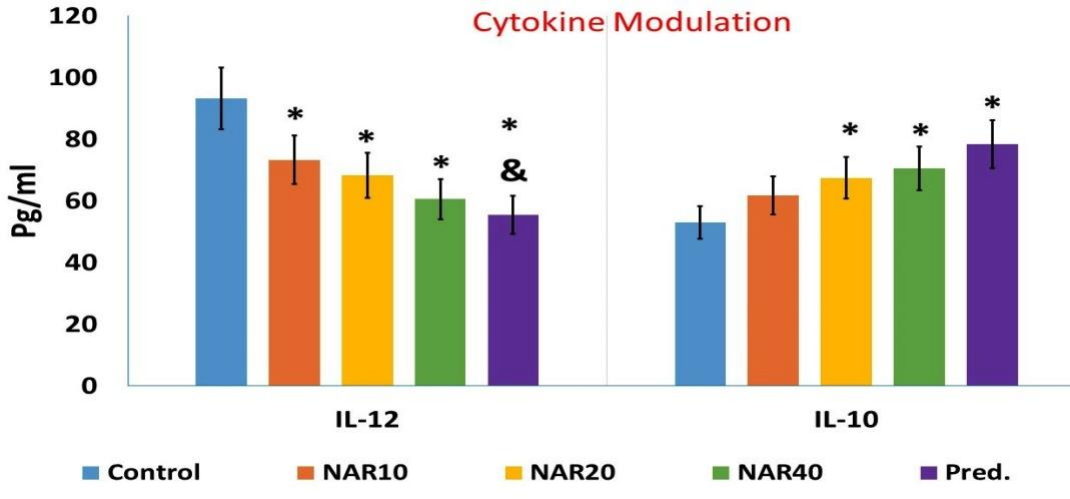
Cytokine modulation of macrophage after challenge with lipopolysaccharide. DThe results are presented as mean±S.D. (* p<0.05 versus control mice, & p<0.05 versus NAR10 mice)

*Ex vivo* performance of peritoneal macrophages obtained from mice administered with naringenin or prednisolone for one month differed significantly from the control group, as shown in Figure 1. Macrophages isolated from the peritoneum of mice administered with naringenin dose-dependently produced less nitric oxide and oxygen-free radicals compared to macrophages isolated from the control mice (Figure 1 A and B). The LPS-stimulated macrophages isolated from mice receiving naringenin secreted lower levels of IL-12 in a dose-independent manner compared to macrophages isolated from the control mice. In contrast, the secretion of IL-10 was considerably increased in LPS-stimulated macrophages isolated from mice administered with 20 and 40 mg/kg naringenin for one month. Conversely, LPS-stimulated macrophages isolated from mice that received 20 and 40 mg/kg of naringenin for one month exhibited a significant increase in IL-10 secretion (Figure 2). 

The dose-dependent administration of naringenin increased macrophage zymosan phagocytosis to a greater extent than macrophages isolated from the control mice (Figure 1 C). Nevertheless, treatment with naringenin at a 40 mg/kg dose increased the phagocytic activity of macrophage more than the phagocytic activity of macrophages isolated from mice treated with prednisolone (Figure 1 C). 

 A one-month treatment with prednisolone produced a similar pattern of changes in macrophage functions as with naringenin treatment (decreased nitric oxide and oxygen-free radicals’ production, lower levels of IL-12 and higher levels of IL-10, and increased phagocytosis compared to macrophages in the control group) (Figure 1 and 2). The changes in nitric oxide and oxygen-free radical production, as well as IL-12 and IL-10 levels following treatment with 40 mg/kg of naringenin were not significantly different from the changes in the same factors following prednisolone treatment (Figure 1 and 2). 

**Naringenin regressed DTH responses in OVA-immunized mice**

**Figure 3:**
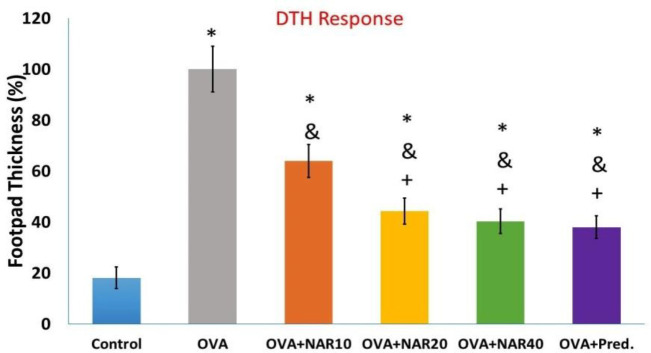
Effects of treatments on DTH response. Ovalbumin-immunized mice treated with naringenin or prednisolone received an SC injection of 50 µl OVA (1 mg/ml in equal volumes of PBS and IFA) into the left hind paw on the twenty-eighth day after the start of the immunization. The same volume of PBS was injected into the right foot pad as a negative control. After 48 hr, DTH reaction was monitored by a digital caliper, as described in materials and methods. The findings are reported as mean±SD. (* p<0.05 versus control mice, & p<0.05 versus OVA immunized mice, + p<0.05 versus OVA+ NAR10 mice)

At the time of DTH evaluation, the mean value for mice immunized only with OVA was considered 100%. According to the collected data, all treatment protocols markedly diminished DTH responses in OVA-immunized mice. In addition, OVA-challenged mice treated with prednisolone had the same effect in reducing DTH as OVA-challenged mice treated with 20 or 40 mg/kg of naringenin (Figure 3). 

**Naringenin demonstrated a dose-dependent decrease in lymphocyte proliferation**

**Figure 4:**
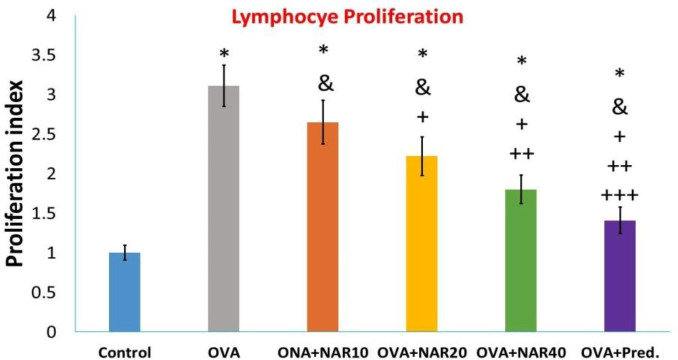
Assessment of *ex vivo *lymphocyte proliferation. Following one month of oral treatment of Balb/c mice with naringenin or prednisolone, splenocytes from OVA-immunized mice were isolated and then cultured in the presence of OVA, as described in material and methods. The data were reported as mean±SD (* p<0.05 versus control mice, & p<0.05 versus OVA immunized mice, + p<0.05 versus OVA+ NAR10 mice, ++ p<0.05 versus OVA+ NAR20 mice, +++p<0.05 versus OVA+ NAR40 mice)

To determine the effects of each drug on specific proliferative responses, harvested splenocytes were cultured in the presence of OVA. As depicted in Figure 3, OVA immunization increased lymphocyte proliferation in the presence of the specific antigen. When challenged *ex vivo* with the specific antigen, OVA-challenged mice administered with naringenin demonstrated a dose-dependent decrease in lymphocyte proliferation. More importantly, lymphocyte proliferation analysis revealed that prednisolone treatment resulted in a greater decrease in splenocyte proliferation than the highest naringenin dos (Figure 4).

**Naringenin modified Th polarizationin OVA-immunized mice**

**Figure 5:**
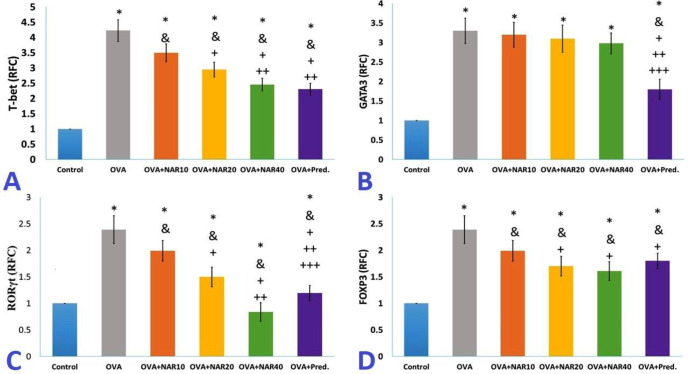
Th polarization changes after treatments. Total RNA from the splenocyte population cultured with OVA or medium was extracted to analyze *Foxp3, T-bet, RORγt,* and *GATA3* expression. The data are presented as mean±SD (* p<0.05 versus control mice, & p<0.05 versus OVA immunized mice, + p<0.05 versus OVA+ NAR10 mice, ++ p<0.05 versus OVA+ NAR20 mice, +++p<0.05 versus OVA+ NAR40 mice)

After one month of treatment with naringenin in mice immunized with OVA, the mRNA expression level of *T-bet* decreased in a dose-independent manner. In addition, one month of prednisolone treatment led to a significant decrease in the mRNA expression level of *T-bet *There was no substantial difference in the decrease in expression of this factor between the immunized mice that received 40 mg/kg of naringenin or 2 mg/kg of prednisolone (Figure 5 A).

The statistical analysis of the expression of *GATA3* demonstrated that treatment with naringenin does not significantly alter the expression of this factor in mice immunized with OVA compared to untreated immunized mice. Nonetheless, prednisolone treatment considerably suppressed the expression of *GATA3* in mice immunized with OVA (Figure 5 B).

*Ex vivo* results indicated that naringenin treatment decreased *RORγt*expression dose-dependently. Also evident was the effect of prednisolone on the expression of *RORγt*. In mice immunized with OVA, one month of administration of 40 mg/kg naringenin reduced the expression of *RORγt* more than one monthof treatment with prednisolone (Figure 5 C).

The administration of 10 mg/kg of naringenin had no significant effect on the expression of *FOXP3*. In mice immunized with OVA, however, treatment with 20 and 40 mg/kg of naringenin or 2 mg/kg of prednisolone reduced the expression of *FOXP3 *compared to untreated mice. There was no statistically significant difference between these three groups (Figure 5 D).

## Discussion

Evidence-based scientific research on plant derivatives provides an exciting starting point for exploring new immunomodulatory agents (Shi et al., 2021).The ultimate goal of the current study was to compare the potential of immune response modification by oral administration of naringenin and prednisolone. At first, the effect of these components on the main activities of peritoneal macrophages was examined. Macrophages play a crucial role in the majority phase of inflammation by removing cell debris, promoting and resolving inflammation, and activating fibrosis (Oishi and Manabe, 2018). M1 or classically activated macrophages possess 

potent antimicrobial and inflammatory properties and they are involved in the pathogenesis of inflammatory diseases (Cutolo et al., 2022). These macrophages produce high levels of reactive oxygen species (ROS) and nitrogen species (such as nitric oxide) and secrete the cytokine IL-12 to promote the development of type I immune responses (Shushtari and Abtahi Froushani, 2017). M2 and alternatively activated macrophages produce fewer proinflammatory substances, such as ROS and NO. Through the secretion of trophic factors and the production of anti-inflammatory cytokines like IL-10, these macrophages play an essential role in reducing inflammation (Cutolo et al., 2022). Due to the phagocytosis of tissue debris (efferocytosis) and M2 macrophages, the phagocytic activity of these macrophages is greater than that of M1 (Bi et al., 2019; Shushtari and Abtahi Froushani, 2017). 

According to the findings of our study, macrophages isolated from the peritoneum of mice treated with naringenin produced dose-dependently lower levels of NO and oxygen-free radicals. This group of macrophages produced less IL-12 than the control group, whereas the production of IL-10 increased significantly in macrophages isolated from mice administered with 20 and 40 mg/kg of naringenin for one month. In addition, their phagocytic activity decreased markedly compared to the control group. These findings point to the development of the anti-inflammatory phenotype (M2 like) in macrophages isolated from the mice administered with naringenin. Recent research has demonstrated that naringenin inhibits the production of proinflammatory cytokines by macrophages by inhibiting NF‑κB activation (Yang et al., 2021). According to another study, naringenin promotes degradation of intracellular cytokines IL-6 and TNF-α through lysosome- and TFEB-dependent mechanisms (Jin et al., 2017). Similar to naringenin, a one-month course of prednisolone led to a similar pattern of macrophage findings. In macrophages that are not stimulated by proinflammatory mediators, it is well established that glucocorticoids can induce gene expression, including anti-inflammatory proteins such as IL-10 or annexin A1, in addition to boosting phagocytic activity (Ehrchen et al., 2019). Our data also revealed that receiving 40 mg/kg of naringenin for one month increased macrophage phagocytic activity more than macrophages isolated from the mice given prednisolone.

The precise balance between T lymphocytes with distinct inflammatory and tolerant functions is essential for generating a protective immune response against pathogens without compromising immune tolerance to self-antigens. CD4^+^ Th lymphocytes play crucial roles in immunoregulatory processes and in shaping the type of immune response (Ahmad et al., 2022; Etemadi et al., 2022). Recent *in vitro* research indicates that naringenin primarily affects CD4^+^ T lymphocytes (Th cells), as opposed to CD8^+^ T lymphocytes (Wang et al., 2018a). In this regard, the focus of the current research was more on Th cells. Th1 cells, mediated by lymphocytes expressing T-bet, protect the organism from the majority of infections and are responsible for perpetuating autoinflammatory and autoimmune responses. While Th2 lymphocytes, mediated by GATA3-expressing lymphocytes, can protect the organism from worm parasites and aid in the resolution of cell-mediated inflammation (Moriyama and Nakamura, 2017). *Ex vivo* data from the current study revealed naringenin affects and reverses Th1 response rather than Th2 response. *In vitro* data demonstrated that naringenin inhibited IFN-γ production by CD4^+^ T cells in a dose-dependent manner (Wang et al., 2018a). It is recognized that a dietary supplement containing 5% naringenin can inhibit the expression of mRNA of Th1-recruiting chemokines such as CXCL10 in the CNS of EAE mice when administered for 30 days (Wang et al., 2018b). In accordance with our findings, prior research indicates that naringenin does not affect the polarization of lymphocytes toward Th2. Flow cytometric analysis revealed that EAE mice fed with naringenin for 30 days had a comparable population of IL-4^+^ CD4^+^ cells to those fed with a control diet (Wang et al., 2018b). Also, according to an *in vitro* study, 80 μMnaringenin did not affect the percentage of IL-4^+^ CD4^+^ cells in the anti-CD3/CD28-activated T cell population (Wang et al., 2018a).

Th17 and Treg lymphocytes are active components of immunity and tolerance formation. The purpose of Th17 cells is to defend the host against extracellular bacteria and fungi (Etemadi et al., 2022; Tuzlak et al., 2021). In immunopathological conditions where Th17 responses are frequently exaggerated, the body’s immune system shifts toward an inflammatory phenotype and attacks healthy tissue. Specific anti-inflammatory mediators are expressed by Treg cells, dampening the excessive effector immune response. While *RORγt* is the master regulator of Th17, *FOXP3* is the master regulator of Treg (Tuzlak et al., 2021). The proinflammatory Th17 and Th9 lymphocytes and their master transcription factors *RORγt* and *PU.1* were downregulated in the lymph nodes and CNS of naringenin-fed EAE C57bl/6 mice (Wang et al., 2018b). The effect of naringenin on the expression of *RORγt* was also evident in our study. *In vitro* data indicated that naringenin could induce iTreg cells in anti-CD3/CD28 activated T cell populations (Wang et al., 2018a). Flow-cytometric analysis of the lymph node cell population of EAE mice administered with 5% naringenin for one month revealed no changes in the Treg cell population (Wang et al., 2018b). An earlier *in vitro* study suggested that relief of immunosuppression induced by regulatory T cells may be the underlying mechanism of metastasis inhibition by naringenin in a metastatic lung cancer model (Qin et al., 2011). The results of this study indicated that doses of 20 and 40 mg/kg of naringenin reduced the expression of *FOXP3* in mice immunized with OVA. The 10 mg/kg dose of naringenin had no discernible effect on the expression of this factor. Notably, these contradictory outcomes were produced under different conditions, making data interpretation challenging.

Overall, naringenin reduced Th1 and Th17 responses in mice immunized with OVA and administered with naringenin. Alterations in lymphocyte polarization induced by naringenin may aid in treating immune-mediated diseases. Recent research has demonstrated that treatment with naringenin after the onset of collagen-induced arthritis can alleviate arthritis symptoms by modulating the levels of the cytokines IFN-γ and IL-17, myeloperoxidase, nitric oxide, and C-reactive protein (Hajizadeh et al., 2021).

In mice immunized with OVA, a one-month treatment with prednisolone inhibited the expression of transcription factors associated with lymphocyte polarization. This occurred even though naringenin did not affect the expression of the *GATA3* factor. Similarly, the 40 mg/kg naringenin dose reduced *RORγt* more effectively than prednisolone. Specifically, Th17 lymphocytes initiate immunopathological conditions and are more pathogenic than Th1 cells (Abtahi Froushani et al., 2014). Therefore, this can be considered an advantage for naringenin.

*In vivo* data revealed that OVA-challenged mice treated with 20 or 40 mg/kg of naringenin or 2 mg/kg of prednisolone for one month exhibited a similar reduction in DTH compared to the control mice. Given that the cooperation of M1-macrophages and Th1-lymphocytes is required to form a classical DTH (Zimecki et al., 2021), these findings make sense in light of prior research. Treatment with naringenin decreased the visceral leishmaniasis burden in BALB/c mice while simultaneously promoting the DTH immune response against the parasite (Kaur et al., 2018).

*Ex vivo* data indicate that the splenocyte proliferation index was lower in the prednisolone-treated group than in splenocytes isolated from the mice administered with the highest dose of naringenin. Recent research suggested that naringenin could inhibit T cell proliferation by inducing T cell cycle arrest at the G0/G1 phase, which was associated with delayed degradation of the cyclin-dependent kinase inhibitor p27kip1 and decreased retinoblastoma protein phosphorylation in activated T lymphocytes (Niu et al., 2018). Additionally, naringenin reversed picryl chloride-induced contact hypersensitivity by inhibiting T lymphocyte proliferation (Fang et al., 2010).

Overall, in many cases, naringenin acted similarly to prednisolone, so that both substances inhibited both the inflammatory function of macrophages and the function of OVA-specific lymphocytes. Less inhibition of lymphocyte proliferation in response to the specific antigen, and further inhibition of Th17 immunopathological lymphocytes, as well as increased capacity clearance and phagocytosis of macrophages, are all advantages of a one-month dose of 40 mg/kg naringenin versus 2 mg/kg prednisolone. All these findings indicate the potential of naringenin as a modifying agent of immune responses. Consequently, naringenin may be beneficial in controlling some immunopathological conditions. However, this is only a preliminary study, and in the future, it will be necessary to conduct more extensive research with higher doses and in various immunopathological conditions. 

## References

[1] Abtahi Froushani SM, Delirezh N, Hobbenaghi R, Mosayebi G (2014). Synergistic effects of atorvastatin and all-trans retinoic acid in ameliorating animal model of multiple sclerosis. Immunol Invest.

[2] Abtahi Froushani SM, Esmaili Gourvarchin Galeh H (2014). New insight into the immunomodulatory mechanisms of tretinoin in NMRI mice. Iran J Basic Med Sci.

[3] Ahmad HI, Jabbar A, Mushtaq N, Javed Z, Hayyat MU, Bashir J, Naseeb I, Abideen ZU, Ahmad N, Chen J (2022). Immune tolerance vs immune resistance: the interaction between host and pathogens in infectious diseases. Front Vet Sci.

[4] Bart VT, Pickering RJ, Taylor PR, Ipseiz N (2021). Macrophage reprogramming for therapy. Immunology.

[5] Bi Y, Chen J, Hu F, Liu J, Li M, Zhao L (2019). M2 macrophages as a potential target for antiatherosclerosis treatment. Neural Plast.

[6] Boechat JL, Chora I, Morais A, Delgado L (2021). The immune response to SARS-CoV-2 and COVID-19 immunopathology - current perspectives. Pulmonology.

[7] Clementi N, Scagnolari C, D'Amore A, Palombi F, Criscuolo E, Frasca F, Pierangeli A, Mancini N, Antonelli G, Clementi M, Carpaneto A, Filippini A (2021). Naringenin is a powerful inhibitor of SARS-CoV-2 infection in vitro. Pharmacol Res.

[8] Cutolo M, Campitiello R, Gotelli E, Soldano S (2022). The Role of M1/M2 macrophage polarization in rheumatoid arthritis synovitis. Front Immunol.

[9] Deenonpoe R, Prayong P, Thippamom N, Meephansan J, Na-Bangchang K (2019). Anti-inflammatory effect of naringin and sericin combination on human peripheral blood mononuclear cells (hPBMCs) from patient with psoriasis. BMC Complement Altern Med.

[10] Ehrchen JM, Roth J, Barczyk-Kahlert K (2019). More than suppression: glucocorticoid action on monocytes and macrophages. Front Immunol.

[11] Erlund I, Meririnne E, Alfthan G, Aro A (2001). Plasma kinetics and urinary excretion of the flavanones naringenin and hesperetin in humans after ingestion of orange juice and grapefruit Juice. J Nutr.

[12] Etemadi S, Abtahi Froushani SM, Hashemi Asl SM, Mahmoudian A (2022). Combined atorvastatin and pentoxifylline in ameliorating inflammation induced by complete Freund’s adjuvant. Inflammopharmacology.

[13] Fang F, Tang Y, Gao Z, Xu Q (2010). A novel regulatory mechanism of naringenin through inhibition of T lymphocyte function in contact hypersensitivity suppression. Biochem Biophys Res Commun.

[14] Froushani SM, Galeh HE (2014). New insight into the immunomodulatory mechanisms of tretinoin in NMRI mice. Iran J Basic Med Sci.

[15] Golbahari S, Abtahi Froushani SM (2019). Synergistic benefits of nicotine and thymol in alleviating experimental rheumatoid arthritis. Life Sci.

[16] Hajizadeh A, Abtahi Froushani SM, Tehrani AA, Azizi S, Bani Hashemi SR (2021). Effects of naringenin on experimentally induced rheumatoid arthritis in Wistar Rats. Arch Razi Inst.

[17] Hosseinzade A, Sadeghi O, Naghdipour Biregani A, Soukhtehzari S, Brandt GS, Esmaillzadeh A (2019). Immunomodulatory effects of flavonoids: Possible induction of T CD4+ regulatory cells through suppression of mTOR pathway signaling activity. Front Immunol.

[18] Jin L, Zeng W, Zhang F, Zhang C, Liang W (2017). Naringenin ameliorates acute inflammation by regulating intracellular cytokine degradation. J Immunol.

[19] Kaur G, Chauhan K, Kaur S (2018). Immunotherapeutic potential of codonopsis clematidea and naringenin against visceral leishmaniasis. Biomed Pharmacother.

[20] Liu K, Wu L, Shi X, Wu F (2016). Protective effect of naringin against ankylosing spondylitis via ossification, inflammation and oxidative stress in mice. Exp Ther Med.

[21] Moriyama M, Nakamura S (2017). Th1/Th2 Immune balance and other T helper subsets in IgG4-Related disease. Curr Top Microbiol Immunol.

[22] Niu X, Sang H, Wang J (2021). Naringenin attenuates experimental autoimmune encephalomyelitis by protecting the intact of blood-brain barrier and controlling inflammatory cell migration. J Nutr Biochem.

[23] Niu X, Wu C, Li M, Zhao Q, Meydani SN, Wang J, Wu D (2018). Naringenin is an inhibitor of T cell effector functions. Front Immunol.

[24] Oishi Y, Manabe I (2018). Macrophages in inflammation, repair and regeneration. Int Immunol.

[25] Panche AN, Diwan AD, Chandra SR (2016). Flavonoids: an overview. J Nutr Sci.

[26] Parandin R, Ghowsi M, Dadbod A (2023). Protective effects of hydroalcoholic extract of Rosa canina L fruit on cyclophosphamide-induced testicular toxicity in mice. Avicenna J Phytomed.

[27] Pineda-Torra I, Gage M, de Juan A, Pello OM (2015). Isolation, culture, and polarization of murine bone marrow-derived and peritoneal macrophages. Methods Mol Biol.

[28] Qin L, Jin L, Lu L, Lu X, Zhang C, Zhang F, Liang W (2011). Naringenin reduces lung metastasis in a breast cancer resection model. Protein Cell.

[29] Rios FJ, Touyz RM, Montezano AC (2017). Isolation and differentiation of murine macrophages. Methods Mol Biol.

[30] Shi J, Weng JH, Mitchison TJ (2021). Immunomodulatory drug discovery from herbal medicines: Insights from organ-specific activity and xenobiotic defenses. Elife.

[31] Shushtari N, Abtahi Froushani SM (2017). Caffeine augments the instruction of anti-inflammatory macrophages by the conditioned medium of mesenchymal stem cells. Cell J.

[32] Sobotková M, Bartůňková J (2019). Current trends in immunosuppressive treatment. Vnitr Lek.

[33] Tungmunnithum D, Thongboonyou A, Pholboon A, Yangsabai A (2018). Flavonoids and other phenolic compounds from medicinal plants for pharmaceutical and medical aspects: An Overview. Medicines (Basel).

[34] Tuzlak S, Dejean AS, Iannacone M, Quintana FJ, Waisman A, Ginhoux F, Korn T, Becher B (2021). Repositioning T(H) cell polarization from single cytokines to complex help. Nat Immunol.

[35] Wang J, Niu X, Wu C, Wu D (2018a). Naringenin modifies the development of lineage-specific effector CD4(+) T cells. Front Immunol.

[36] Wang J, Qi Y, Niu X, Tang H, Meydani SN, Wu D (2018b). Dietary naringenin supplementation attenuates experimental autoimmune encephalomyelitis by modulating autoimmune inflammatory responses in mice. J Nutr Biochem.

[37] Yang J, Liu L, Li M, Huang X, Yang H, Li K (2021). Naringenin inhibits pro‑inflammatory cytokine production in macrophages through inducing MT1G to suppress the activation of NF‑κB. Mol Immunol.

[38] Zimecki M, Kruzel ML, Hwang SA, Wilk KM, Actor JK (2021). Lactoferrin as an adjuvant for the generation of delayed type hypersensitivity to orally administered antigen. Ann Clin Lab Sci.

